# A 300 bp non-coding repeat sequence has co-evolved with the *ced-2/CRKII-set-23/SETMAR* genes of nematodes

**DOI:** 10.17912/micropub.biology.001845

**Published:** 2025-10-20

**Authors:** Yeshaswi Pulijala, Victoria Brown, Martha Soto

**Affiliations:** 1 Pathology and Laboratory Medicine, Rutgers, The State University of New Jersey, New Brunswick, New Jersey, United States

## Abstract

Highly conserved non-coding repeats may encode sequences with regulatory functions. We report a ~300 nucleotide non-coding repeat found four times in *C. elegans ,* and exclusively at the *ced-2 /CRKII- set-23 /SETMAR* operon. Comparative analysis across nematodes, including *C. briggsae , C. brenneri* , and distantly related *B. malayi* , shows that these repeats are conserved at all *ced-2 /CRKII- set-23 /SETMAR* loci. Furthermore, between *C. elegans* and *B. malayi* these repeats are more highly conserved than the CED-2 /CRKII coding sequences. These findings suggest the repeats are conserved regulatory modules coordinating CED-2 and SET-23 expression. Supporting this idea, the repeats partially resemble miRNA precursors.

**Figure 1. CED-2/CRKII- SET-23/SETMAR repeat characterization:**
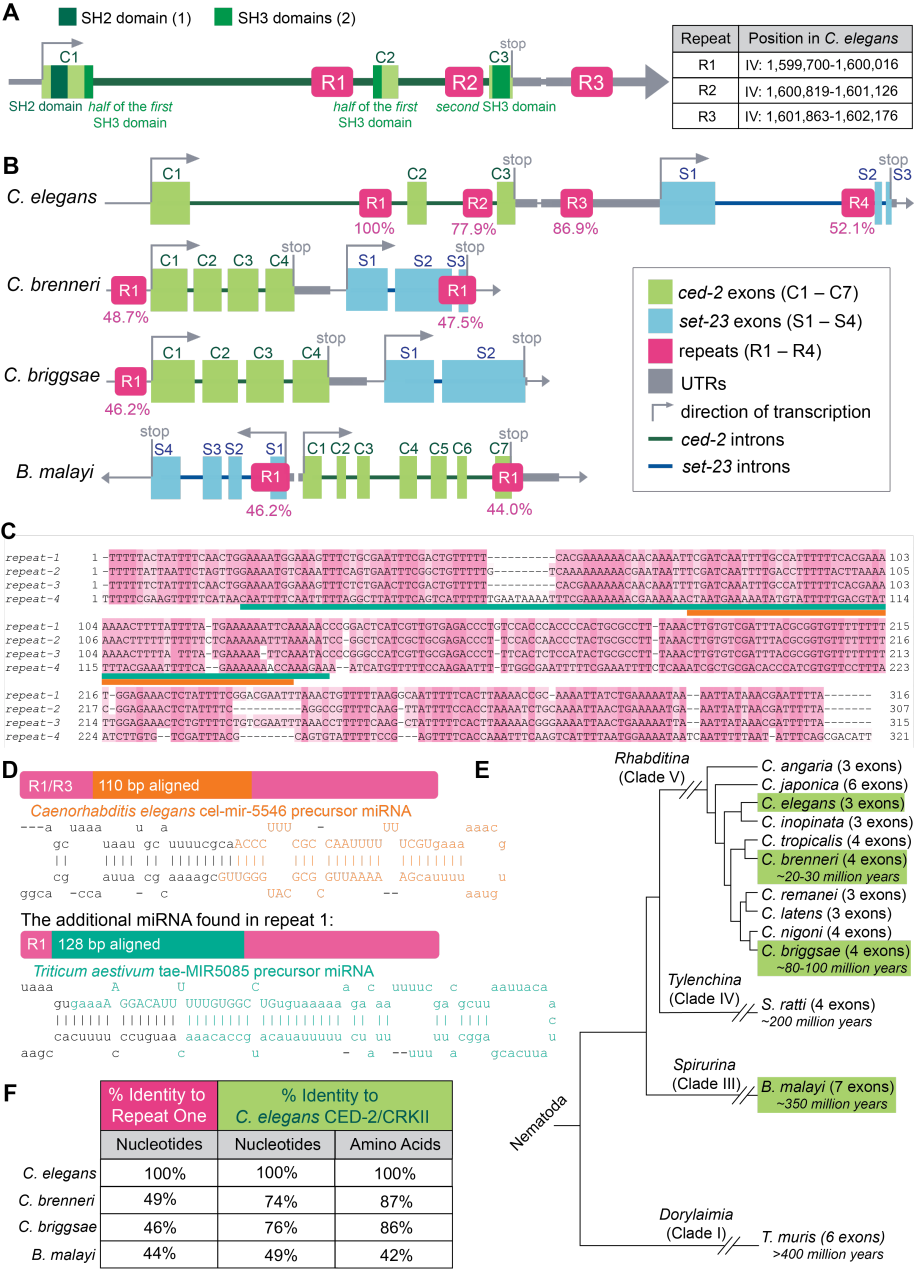
**A.** The CED-2 /CRKII gene in *C. elegans* has three exons (C1, C2, C3). Exon 1 (C1) contains an SH2 domain and the first half of the first SH3 domain. Exon 2 (C2) contains the rest of the first SH3. Exon 3 (C3) contains the second SH3 domain. Repeat sequences, R1, R2 and R3, are found in ced-2 introns and 3' region. A table of exact genomic coordinates for these conserved repeats in the *C. elegans* genome has been provided. **B.** Alignment of *C. elegans ced-2* /CRKII (green), *set-23* /SETMAR (blue), and repeat sequences (pink) to other nematode species. Repeat sequence identity percent (%) in relation to repeat 1 (R1) of *C. elegans .* **C.** Alignment of repeats 1-4 of *C. elegans* to each other. Darker pink indicates identity, lighter pink indicates similarity. Orange and teal bars show where the miRNAs shown in Fig. 1D align to the repeats. **D.** R1 and R3 align to known miRNAs, shown as stem-loop structures. **E.** Phylogenetic analysis of CED-2 exon organization and distance in million years from *C. elegans .* See Methods for sources for dates cited. **F.** Comparison of conservation of repeat sequence identity, vs. CED-2 /CRKII coding sequence identity, across Nematoda species.

## Description

Approximately 2% of the transcribed RNA molecules in the human genome encode proteins (Park et al. 2022). The remaining RNA transcripts are known as non-coding RNAs (ncRNAs). Previously believed to be “junk,” i.e. lacking function, these ncRNAs, including long, non-coding RNAs (lncRNAs), have since been implicated in various biological processes, including epigenetic regulation, chromatin remodeling, organ/tissue development, among others (George et al. 2024). lncRNAs, loosely defined as transcripts longer than 200 nucleotides, are now implicated in human diseases, including cancer (Liu et al. 2016; Sun et al. 2016). Growing evidence suggests that their dysregulation, whether through inappropriate activation, loss of repression, or altered expression patterns, can endow them with oncogenic properties and drive malignant transformation (Huang et al. 2021; Li et al. 2017). lncRNAs are repeat-rich, allowing them to bind chromatin for chromatin remodeling and alternative splicing (Hadjiargyrou and Delihas 2013; Creamer et al. 2021; Yap et al. 2018). For example, the ~300 bp Alu elements, a family of repetitive elements exclusive to primate genomes, are embedded within lncRNAs to form duplexes with complementary Alu elements for post-transcriptional mRNA decay (Gong and Maquat 2011). Repetitive DNA sequences (DNA repeats) account for ~50% of the human genome and are necessary for driving evolution and gene regulation; moreover, their dysregulation is linked to various diseases, including genetic disorders and cancers (Liao et al. 2023).

We noticed that three non-coding regions in the *ced-2* locus have high sequence similarity to each other, but not to other sequences in the *C. elegans* genome. The CED-2 /CRKII protein consists of one SH2 motif and two SH3 motifs. This SH2-SH3-SH3 organization is conserved from *C. elegan* s to humans (Kang et al. 2011). The three repeats in *C. elegans* reside around two exons that contain part of the first SH3 domain and the second SH3 domain of CED-2 /CRKII ( Fig. 1A ). Aligning the three repeats to each other showed that the two outside repeats, numbered R1 and R3 ( Fig. 1A ), have the highest sequence identity, 86.9% over 314 nucleotides. Repeat #2 (R2), which lies between repeat R1 and R3 and between exons 2 and 3, shows 77.9% identity to R1 over 307 nucleotides. Further sequence analysis revealed the presence of another repeat (R4) found in the *set-23* gene, located adjacent to *ced-2* (Fig 1B). SET-23 is the SETMAR (SET domain and mariner transposase fusion gene) ortholog of *C. elegans ,* thus it is predicted to regulate chromatin remodeling and methylation (Sternberg et al. 2024; Tellier 2021). *ced-2* and *set-23* appear to be in an operon, with transcription starting at *ced-2* and continuing into *set-23* . R4 resides in the intron between the two *set-23* exons, S1 and S2, and partially overlaps with S2 ( Fig. 1B ). Alignment of the four repeats revealed high similarity (light pink) and high identity (dark pink) all throughout the 307-323 nucleotide-long repeats ( Fig. 1C ). Comparing R4 to R1 shows 52.1% identity over 282 nucleotides ( Fig. 1B- C).

To assess whether the repeats were transposon-derived, each sequence was entered into the RepBase Database. RepBase (CENSOR) (Kohany et al. 2006) identified weak matches (20.9% identity Chapev-34_HM vs. R1, 15.4% identity EnSpm-1_CGi vs. R2, 29.6% identity Gypsy-14_TeGr-I and Polinton-10_SM combined vs. R3). These levels are comparable to background similarity found in random *C. elegans* sequences relative to any transposons (e.g., 17.5% for *ced-2* exon 1, 34.4% for a non-coding *ced-2* region, 23.8% for *ced-5* exon 2, and 47.6% for the *ced-5* intronic region between exons 6 and 7). Thus, the analysis does not support a strong relationship between these repeats and transposons.

Using miRbase (Kozomara et al. 2019; Kozomara et al. 2011; Kozomara et al. 2014), we explored whether these repeats aligned to any precursor or mature miRNA. Both R1 and R3 showed similarity to the *Caenorhabditis elegans* cel-mir-5546 precursor miRNA, and both aligned to a 110-base pair region in this stem-loop ( Fig. 1D ). Additionally, R1 showed similarity to *Triticum aestivum* tae-MIR5085 precursor miRNA, over 128 bp ( Fig. 1D ). Given that ~17.5% of miRNAs are embedded within lncRNAs (He et al. 2021; Dhir et al. 2015; Quinn and Chang 2016), it is perhaps unsurprising that roughly one-third of each repeat's length aligns to known miRNAs. Searching with Rfam (Ontiveros-Palacios et al. 2024) also identified similarity to miRNAs and other non-coding RNAs. Overall, this suggested that these repeats may function as miRNA regulators of *ced-2* and *set-23* . Notably, the regions of alignment overlap, with the *C. elegans* cel-mir-5546 precursor miRNA falling within the broader region corresponding to the *T. aestivum* tae-MIR5085 precursor miRNA ( Fig. 1C ).

We explored whether these repeats are associated with the CED-2 /CRKII locus in other nematodes. Aligning *C. elegans* *ced-2* genomic DNA to closely related ( *C. brenneri* and *C. briggsae* ) and more distantly related *(B* ***.*** *malayi* ) nematodes (Carlton et al. 2022; Gupta and Sternberg 2003) ( Fig. 1B, E) showed that these species of *Caenorhabditis* contain these repeats, and that they are exclusively found within a region encompassing both *ced* - *2* and *set-23* . Even in the distantly related nematode species, *B. malayi* , repeats were found in a region encompassing both *ced-2* and *set-23* genes ( Fig. 1B ), which may indicate these nucleotides support *ced-2* and *set-23* regulation or function. Comparing species from other nematode clades with available sequence, *T. muris* (Clade I) and *S. ratti* (Clade IV), both contained *ced-2* /CRKII orthologs with 51% identify to R1. *set-23* /SETMAR was on the same chromosome ( *T. muris* ) with 49% identity to R1, and on a different chromosome ( *S. ratti* ) with 52% and 47% identify to R1 and R4, respectively.

Comparing the *ced-2* /CRKII gene across species illustrated that while these genes are orthologous, their structures can differ substantially in exon organization. Comparative alignments of exon distribution across species revealed that the number of CED-2 exons can vary widely, from as few as 3 in *C. elegans* , to as many as 7 in *Brugia malayi* , a parasitic nematode that diverged from *C. elegans* roughly 350 million years ago ( Fig. 1B and 1E) (Ghedin et al. 2007). Gene length also varies in unexpected ways. In some species, the combined length of the orthologous CED-2 /CRKII and SET-23 genes is shorter than the *C. elegans* *ced-2* genomic sequence alone. This suggests substantial differences in noncoding sequence content or intron size, possibly reflecting lineage-specific regulatory or structural constraints.

The *C. elegans* *ced-2* locus itself has an intriguing exon–intron organization. Notably, its first SH3 domain is split between exon 1 and exon 2, an arrangement that complicates hypotheses about domain evolution. This split organization argues against the idea that the conserved repeats within *ced-2* support a straightforward SH3 domain duplication ( Fig. 1A ). Because the *ced-2* and *set-23* genes dramatically differ in exon organization across species, the presence of conserved repeats at the *ced-2 / set-23* locus is striking.

The *C. elegans* repeats show 50%-86.9% identity to each other when R1 is aligned to the 3 other repeats ( Fig. 1B ). We were interested in comparing the conservation of the repeats to the conservation of the CED-2 /CRKII coding sequence. We aligned *C. elegans* R1 to one repeat in *C. brenneri , C. briggsae ,* and *B. malayi* , and also aligned the CED-2 /CRKII coding sequence, at the level of nucleotides and amino acids, among these four nematodes ( Fig. 1F ). CED-2 /CRKII shared only 49% nucleotide and 42% amino acid identity between *C. elegans* and *B. malayi* , while R1 retained 53% identity. Thus, these non-coding repeats are more conserved across 350 million years of evolution than the associated coding sequences ( Fig. 1F ) (Ghedin et al. 2007).

To test if the repeats are conserved beyond nematodes, we aligned CED-2 /CRKII and SET-23 /SETMAR to *Drosophila melanogaster* . In *D. melanogaster* , CED-2 /CRKII is on Chromosome 4, and SET-23 /SETMAR on Chromosome 3. However, both genes contained the repeats: CRK/CRKII aligned to R4 (48% identity over 323 nucleotides), while CG4565/SETMAR aligned to R1 (49% identity over 290 nucleotides) and R4 (44% identity over 289 nucleotides). This suggested that CED-2 /CRKII and SETMAR may be regulated by these conserved sequences beyond nematodes.

Taken together, the conserved proximity of *ced-2* and *set-23* in most nematodes, and the highly conserved repeats, suggest that these elements may not be passive genomic features but active regulatory modules influencing the regulation of both genes across nematode species. Such a mechanism would be consistent with their shared operonic context and could help coordinate the transcriptional regulation of the *ced-2* and *set-23* genes despite their structural divergence across species.

Repetitive sequences may possess functional relevance that has yet to be examined. Clustered regularly interspaced short palindromic repeat (CRISPR)-Cas systems are well-known forms of acquired immunity found in archaea and bacteria, first identified 30 years ago by the Nakata Lab in *Escherichia coli* while investigating the gene responsible for isozyme conversion of alkaline phosphatase (Ishino et al. 1987). Ishino mentioned repeats with a conserved nucleotide sequence, but could not report biological significance. As more genes associated with CRISPR were discovered and designated as *Cas* (CRISPR-associated) genes, the puzzle pieces fell into place (Griffiths-Jones 2004; Mojica et al. 2005). As DNA sequencing techniques improved, it became clear these sequences were functionally linked across bacteria and archaea. Today, CRISPR-Cas systems are regarded as among the most reliable tools for genome editing and engineering. In this spirit, we report this unusual example of co-evolution of coding and non-coding sequences.

## Methods

**Repeat identification.** An 18-bp primer sequence (5′ ttgtgtcgatttacgcgg 3′) was mapped to the *ced-2* gene of the N2 Bristol strain of *Caenorhabditis elegans* , and yielded three matches within the gene. The genomic *ced-2* region, obtained from WormBase, was aligned to the 18 bp sequence of the primer, using NCBI Nucleotide BLAST with the setting “Somewhat similar sequences (blastn)”. Unselecting the “Short queries” adjustment option resulted in 3 perfect matches (Altschul et al. 1997). The 18 bp matched regions, along with 500 bp of upstream and downstream flanking sequence, created ~1000 bp regions that were aligned using Clustal Omega (Sievers et al. 2011; Goujon et al. 2010; Madeira et al. 2024). The alignments revealed that each 18-bp repeat was embedded within a 43 bp identical sequence present at all three loci. Across the ~1000 bp alignments, approximately 300 bp were found within a highly conserved region (>75% sequence identity). The conserved region spanned 316 bp for repeat 1 (IV:1,599,700-1,600,016), 307 bp for repeat 2 (IV:1,600,819-1,601,126), and 313 bp for repeat 3 (IV:1,601,863-1,602,176) (labeled R1, R2, and R3, Fig. 1A, B, C).

Next, we found the nematode relatives with characterized genomes. Using NCBI, WormBase, Alliance, and JBrowse, *C. elegans* CED-2 /CRKII was aligned to CRKII orthologs from other nematode species, *C. brenneri* , *C. briggsae* , and *B. malayi* . NCBI, WormBase, and Alliance were used to find the orthologous sequences (Sternberg 2024; Diesh et al. 2023). JBrowse was used to visualize the coding/non-coding sequences in these different species, as well as to characterize the genes around their respective CED-2 /CRKII loci.

After aligning these ~300 bp sequences to each other, we found that repeat 1 has 77.9% identity to repeat 2, 86.9% identity to repeat 3, and that repeat 3 has 76.6% identity to repeat 2. Since repeat 1 has the highest homology to either repeat, we chose to use repeat 1 to search for repeats within other species. Repeat 1 was aligned to the orthologous CED-2 /CRKII sequence to find where the repeat lies in the other species. The region where the repeat aligned was then used to calculate the percent (%) identity ( Fig. 1D ). Clustal Omega was used to align the repeats and coding sequences. Repeat 1 was searched against the complete genome using BLAST, which produced no additional significant hits. The sequence was subsequently aligned to the *ced-2* locus, leading to the identification of corresponding repeat regions for comparison across species.

**Repeat Characterization.** To check if the repeats were related to transposons, we used RepBase (CENSOR)'s repeat masking service and submitted the individual repeat sequences to be searched against “All.” This allowed us to search our repeats against all species in their collection of repeats and orthologous regions (Kohany et al. 2006). To test if part of our repeated sequence contains miRNAs, we used miRBase and searched each repeat sequence individually (Kozomara et al. 2019; Kozomara and Griffiths-Jones 2011; Kozomara and Griffiths-Jones 2014; Griffiths-Jones et al. 2004; Griffiths-Jones et al. 2008; Griffiths-Jones et al. 2006; Memar et al. 2019). To further analyze the repeats, we used Rfam (Ontiveros-Palacios et al. 2025) and this program identified some of the same miRNAs shown in Fig. 1D, also aligning to a similar regions as shown in Fig. 1C plus other non-coding RNAs like the long non-coding RNA *linc-128* .

The phylogenetic tree, Fig. 1E, was based on information from Carlton et al. 2022. The years of divergence between *C. elegans* and *C. brenneri* , *C. briggsae* , *B. malayi* , and *T. muris* were based on Memar et al. 2019, Gupta and Sternberg 2003, Ghedin et al. 2007, and Mitreva et al. 2011, respectively.
